# Hepatitis E virus infection in kidney transplant recipients: a pilot cross-sectional study of serological markers and allograft function in Northern Serbia

**DOI:** 10.3389/fneph.2026.1864349

**Published:** 2026-07-29

**Authors:** Sonja B. Golubović, Jelena D. Stojčević Maletić, Lada V. Petrović, Tamaš R. Petrović, Violeta V. Knežević, Maja S. Ružić

**Affiliations:** 1 Faculty of Medicine, University of Novi Sad, Novi Sad, Serbia; 2 Clinic for Nephrology and Clinical Immunology, University Clinical Center of Vojvodina, Novi Sad, Serbia; 3 Center for Laboratory Diagnostics, University Clinical Center of Vojvodina, Novi Sad, Serbia; 4 Scientific Veterinary Institute “Novi Sad”, Novi Sad, Serbia; 5 Clinic for Infectious Diseases, University Clinical Centre of Vojvodina, Novi Sad, Serbia

**Keywords:** extrahepatic manifestations, graft dysfunction, hepatitis E virus, HEV seroprevalence, immunosuppression, kidney transplantation

## Abstract

**Background:**

Hepatitis E virus (HEV) is an emerging cause of chronic hepatitis and extrahepatic disease in solid organ transplant recipients. Data on kidney transplant populations, particularly in endemic regions, remain limited. This pilot study aimed to quantify the prevalence of HEV exposure and active infection in kidney transplant recipients and examine associations between HEV markers and allograft function.

**Methods:**

In this single-centre cross-sectional pilot study, 25 adult kidney transplant recipients (mean age 48.9 ± 14.4 years, median 13.0 years post-transplant) with available HEV serology and/or RNA testing were included. Clinical data, liver enzymes, estimated glomerular filtration rate (eGFR, mL/min/1.73 m²), and urinary albumin-to-creatinine ratio were obtained from medical records; allograft function was categorised as better versus worsening based on longitudinal clinical and laboratory trends documented in medical records. HEV exposure and active infection were assessed using anti-HEV IgG and IgM antibodies (Euroimmun ELISA; reactive ≥ 1.1, borderline 0.8–1.1, negative < 0.8) and HEV RNA in serum and stool.

**Results:**

Anti-HEV antibodies were present in 19/25 (76%) patients; HEV RNA was detected in 2/25 (8%) patients, with concurrent serum and stool positivity confirmed in both cases. eGFR data were available in 15 patients (60%). IgG serostatus was not associated with eGFR differences, but IgM-positive recipients had significantly lower eGFR (median 35.5 vs 57.5 mL/min/1.73 m²; p = 0.018; Cliff’s δ = 0.74) and higher liver enzyme activity. In an exploratory regression model (n = 15), anti-HEV IgM positivity was associated with approximately 29.8 mL/min/1.73 m² lower eGFR (R² = 0.50), though this estimate is hypothesis-generating given the small sample. Anti-HEV IgM was the strongest individual predictor of worsening graft function (OR = 3.00; 95% CI 0.37–24.5).

**Conclusions:**

In this endemic setting, kidney transplant recipients showed high HEV seroprevalence and an exploratory association between IgM positivity and impaired allograft function. These hypothesis-generating findings support targeted HEV evaluation—including RNA testing—in kidney transplant recipients with unexplained graft dysfunction or mild transaminase elevation in an endemic South-Eastern European setting. Larger longitudinal studies with kidney biopsies are needed to define the renal impact of HEV and determine whether anti-HEV IgM may serve as a risk-stratification marker.

## 1 Introduction

Hepatitis E virus (HEV) infection has recently been identified as an emerging problem in most developed areas, and in some countries such as the United Kingdom, it is now the leading cause of acute viral hepatitis (1, 2). This infection is usually self-limiting in immunocompetent individuals; however, in immunocompromised hosts, particularly those who undergo solid organ transplantation (SOT) and stem cell transplantation, it can lead to chronic disease and requires extensive treatment (3). The prevalence of chronic HEV infection among SOT recipients varies from 0.67% in the United Kingdom to 3.2% in small cohorts of lung transplant recipients (4, 5).

Moreover, persistent HEV infection is frequently underdiagnosed because of its typically asymptomatic or oligosymptomatic presentation and only mild to modest elevations in hepatic transaminases, which are often attributed to other etiologies such as infections, malignancies, graft-versus-host disease (GvHD), or drug-induced liver injury (6). This can delay appropriate diagnosis and treatment, and without timely recognition and management, ongoing viral replication may lead to progressive hepatic fibrosis and, in up to 15% of cases, progression to cirrhosis (7, 8). However, most available data originate from liver transplant patients in developed countries such as France, the USA, and Japan, while reports on HEV infection specifically in kidney transplant recipients remain scarce (4, 5).

HEV infections can also lead to extrahepatic manifestations, including neurological symptoms and various glomerulopathies such as membranoproliferative, membranous, or mesangioproliferative glomerulonephritis (9, 10). Glomerulonephritis and other immune-mediated injuries are among the important causes of graft dysfunction and failure in kidney transplant recipients, which provides a rationale to explore whether HEV infection might act as a potential trigger or modifier of glomerular disease in this population (11). Several single-centre cohorts from other regions, including Iran, Japan, Brazil, and Germany, have reported HEV exposure and, in some cases, chronic infection among renal transplant recipients, indicating that HEV is a relevant issue in kidney transplantation beyond our setting. However, data from South-Eastern Europe remain scarce, and little is known about HEV seroepidemiology and its potential association with kidney allograft function in Serbia. Therefore, we conducted a cross-sectional study at a single transplant centre with the aim of investigating the prevalence of HEV infection, to describe the clinical and biochemical characteristics of kidney transplant recipients with evidence of HEV exposure, and to evaluate the association between HEV serological markers and kidney allograft function.

## 2 Materials and methods

### 2.1 Study design and population

We conducted a cross-sectional pilot study of adult kidney transplant recipients who were treated and followed up at the University Clinical Centre of Vojvodina, Clinic for Nephrology and Clinical Immunology, and Clinic for Infectious Diseases at the time of the study. This study was approved by the Ethics Committee of the University Clinical Centre of Vojvodina. Given the retrospective nature of data extraction from existing medical records, written informed consent was not required under national legislation and institutional requirements; all participants were nonetheless enrolled during routine clinical follow-up and were fully informed of the study’s purpose. This study was conducted in accordance with the ethical principles of the Declaration of Helsinki for medical research involving human participants and identifiable human data. All consecutive kidney transplant recipients with available anti-HEV serology and/or HEV RNA testing results obtained from March 2025 to December 2025 were included in the study. A total of 25 kidney transplant recipients fulfilled the inclusion criteria and were included in the final analysis. For this transplantation study, no organs or tissues were obtained from prisoners or other institutionalised individuals. All transplanted organs were procured through a local hospital transplant program following applicable national laws and international ethical standards.

### 2.2 Data collection

Demographic data (age and sex), time since kidney transplantation, etiology of end-stage kidney disease (ESKD), current immunosuppressive regimen, and clinician-rated assessment of graft function were collected from medical records. Immunosuppressive regimen data, including the type of calcineurin inhibitor (tacrolimus or cyclosporine), use of everolimus, mycophenolic acid compounds (mycophenolate mofetil or enteric-coated mycophenolate sodium), prednisolone, and available drug trough levels at the time of HEV testing, were collected for all patients and are summarised in **Table 1**. Laboratory parameters included eGFR (mL/min/1.73 m², calculated using the CKD-EPI equation), liver enzymes (alanine aminotransferase [ALT], aspartate aminotransferase [AST], gamma-glutamyl transferase [GGT], and alkaline phosphatase [ALP]), and urinary protein excretion expressed as the urinary albumin-to-creatinine ratio (UACR). Missing eGFR, UACR, and liver enzyme data reflected the retrospective design and variability in test ordering at routine clinical visits; data were not missing by design, and no systematic difference between patients with and without available measurements was identified, though this limitation is acknowledged. Bilirubin and synthetic liver function parameters (albumin, INR) were not systematically recorded at the time-point of HEV testing in this retrospective cohort and are therefore not reported. All patients underwent routine pre- and post-transplant screening for HBV (HBsAg, anti-HBc, anti-HBs) and HCV (anti-HCV); no patient had active HBV infection documented at the time of the study. One patient had concurrent HCV/HEV co-infection, as detailed in the Results. Systematic exclusion of HAV and HDV co-infection was not performed as part of this retrospective study, which is acknowledged as a limitation. CMV and EBV monitoring is part of routine post-transplant surveillance at our centre; however, CMV and EBV PCR results were not systematically extracted from the medical records and cannot be reported for the full cohort. Stool specimens were analysed for HEV RNA as described in the HEV RNA from Stool section below.

**Table 1:** Baseline characteristics, HEV prevalence, and immunosuppressive therapy.

Variable	Total (N = 25)
Age, years, mean ± SD	48.9 ± 14.4
Female sex, n (%)	12 (48)
Time since Tx, years, median (range)	13.0 (2–31)
eGFR, mL/min/1.73 m², median (range)*	51.1 (13.2–89.0)
ALT, U/L, median (range)†	19.0 (9–193)
AST, U/L, median (range)†	22.0 (15–104)
GGT, U/L, median (range)†	26.5 (11–145)
ALP, U/L, median (range)†	95.0 (58–232)
UACR, mg/g, median (range)‡	3.5 (0.3–236.9)
HEV IgG positive, n (%)	10 (40)
HEV IgG borderline, n (%)	9 (36)
HEV IgM positive, n (%)	8 (32)
HEV PCR positive (serum or stool), n (%)	2 (8)
Graft function worsening, n (%)	11/18 (61)

Immunosuppressive therapy (**Table 1**, continued): Tacrolimus-based regimens were used in 18/25 patients (72%; median trough level 5.8 ng/mL, range 3.0–13.7, n = 17); cyclosporine-based regimens in 3/25 (12%; median trough 56.0 ng/mL, range 45.4–120.0, n = 5); everolimus in 5/25 (20%; median trough 4.2 ng/mL, range 3.1–5.2, n = 6). Any mycophenolic acid compound (mycophenolate mofetil or enteric-coated mycophenolate sodium) was used in 19/25 patients (76%), and prednisolone in 24/25 (96%). Some patients received combination regimens (e.g., tacrolimus + everolimus). Trough levels are reported for patients with available measurements at the time of HEV testing.

*n = 15; †n = 24; ‡n = 21.

### 2.3 HEV testing

HEV serology comprised anti-HEV IgM and anti-HEV IgG antibodies, including semi-quantitative IgG titres using an automated ELISA analyser Euroimmun I-2P (Euroimmun, Germany) using commercial reagent kits from Euroimmun (Germany). Results were classified as reactive (ratio ≥ 1.1), borderline (0.8–1.1), or negative (< 0.8) per the manufacturer’s specifications. The proportion of borderline IgG results (9/25; 36%) exceeds what is typically reported in immunocompetent populations, most likely reflecting attenuated humoral immune responses under chronic immunosuppression, where impaired antibody production and blunted seroconversion are well recognised. Borderline results were analysed as a separate category in three-group IgG analyses and excluded in the sensitivity analysis; primary binary comparisons classified borderline as non-positive. Four patients demonstrated isolated anti-HEV IgM positivity (IgM positive, IgG negative or borderline). In immunosuppressed hosts, isolated IgM positivity may represent early acute infection prior to IgG class-switch, an atypical serological response with impaired seroconversion, or—less commonly—a false-positive result; HEV RNA testing was performed concurrently in all patients. Confirmatory follow-up serology was not available for all IgM-positive patients, which is acknowledged as a limitation. Viral RNA was isolated from blood using an automated extractor GeneRotex 96 (Tianlong, China) with a Viral DNA and RNA extraction kit (Tianlong, China). RNA was detected using the RealStar HEV RT-PCR Kit 2.0 (Altona Diagnostics, Germany; lower limit of detection 17 IU/mL).

### 2.4 HEV RNA from Stool

HEV was detected in stool samples (~1 g) homogenised in 1 mL of sterile phosphate-buffered saline (PBS) using molecular diagnostic methods. Genomic HEV RNA was extracted from the supernatants of homogenized stool samples after centrifugation using the commercial IndiSpin Pathogen Kit (Indical Bioscience GmbH, Germany) according to the manufacturer’s instructions. Before extraction, 3 µL of the extraction control (Red 500 reactions ref: MDX028, Meridian Bioscience), which served at the same time as the internal control later in qPCR reaction, was added to each sample (0.2 mL used for extraction). TaqMan-based one-step duplex reverse transcription real-time PCR (RT-qPCR) was conducted using the commercial kit RNA UltraSense One-Step Quantitative RT-PCR System (Invitrogen, Thermo Fisher Scientific USA), with the primers and probes targeting the highly conserved ORF3 region of the HEV genome and thermal profile described by Jothikumar et al. (2006). Briefly, RT-qPCR was performed in 20 μL reaction volumes comprised of 5 μL of 5× Reaction Mix, 0.5 μM of forward primer HEV-F (5’-GGTGGTTTCTGGGGTGAC-3’), 0.5 μM of reverse primer HEV-R (5’-AGGGGTTGGTTGGATGAA-3’), 0.25 μM of HEV-P (5’-6-FAM-TGATTCTCAGCCCTTCGC-BHQ1-3’), 0.8 of IC-internal control mix (MDX028, Meridian Bioscience), and 5 μL of RNA sample. The thermocycling conditions performed on a 7500 Fast Real-Time PCR System (Applied Biosystems, ThermoFisher Scientific, USA) were 15 min at 50 °C, 2 min at 95 °C, followed by 45 PCR cycles of 15 s at 95 °C, annealing and extension for 20 s at 55 °C, and 35 s at 72 °C.

### 2.5 Definitions

Kidney allograft function was assessed using two complementary but separately defined variables. First, eGFR (mL/min/1.73 m², CKD-EPI) served as a continuous quantitative measure of graft function at the time of HEV testing; no dichotomous “worsening eGFR” variable was derived from eGFR values, and eGFR was analysed only as a continuous variable throughout. Second, graft function was categorised qualitatively as “better” versus “worsening” on the basis of the treating nephrologist’s documented longitudinal assessment of serum creatinine and eGFR trends over the preceding 12 months, integrated with the overall clinical course, rather than any single pre-specified numeric eGFR threshold. This categorical rating therefore represented a global clinical judgement recorded in the medical record and was not a mechanical function of the individual eGFR value available for the present cross-sectional analysis. Because the categorical outcome depended on a documented clinician assessment, patients for whom no definitive categorical rating had been recorded were excluded from the graft-function category analyses only; wherever an eGFR measurement was available, these patients were retained in the continuous eGFR analyses. The absence of a documented rating reflected incomplete retrospective documentation—most often insufficient longitudinal creatinine/eGFR data to support a definitive categorical assessment—and was explicitly not interpreted as evidence of stable graft function. Unrated patients were not assumed to be stable, and no imputation of graft-function category was performed. We acknowledge that excluding unrated patients from the categorical analyses, together with the retrospective and non-blinded nature of the rating, may introduce selection and ascertainment bias; this is addressed in the Limitations. Clinicians were not blinded to HEV serological status at the time of assessment, which represents a further potential source of ascertainment bias. Underlying kidney disease was grouped according to the primary etiology of ESKD (glomerulonephritis, diabetic nephropathy, IgA nephropathy, vesicoureteral reflux, systemic lupus erythematosus, and polycystic kidney disease).

### 2.6 Statistical analysis

Continuous variables are expressed as mean ± standard deviation (SD) or median and range, as appropriate, whereas categorical variables are summarised as absolute and relative frequencies. Due to the small sample size and skewed distributions, non-parametric tests were applied. Three-group comparisons across IgG categories (positive, borderline, and negative) were performed using the Kruskal–Wallis test for continuous variables. Binary group comparisons between HEV serostatus (anti-HEV IgG positive vs. non-positive; anti-HEV IgM positive vs. non-positive) and clinical or biochemical parameters were performed using the Mann–Whitney U test for continuous variables and Fisher’s exact test for categorical variables. The correlation between HEV IgG titres and laboratory parameters, as well as between time from transplantation and HEV serostatus, was assessed using Spearman’s rank correlation coefficient. Effect sizes for key Mann–Whitney comparisons were quantified using Cliff’s delta (δ) and interpreted as small (|δ| ≈ 0.15), medium (|δ| ≈ 0.33), or large (|δ| ≥ 0.47). Given the limited sample size, all multivariable modelling was strictly exploratory and hypothesis-generating. Univariate logistic regression was performed with worsening graft function as the dependent variable, and selected predictors of interest (anti-HEV IgM, anti-HEV IgG, time since transplantation, age) were entered individually; odds ratios are reported with 95% confidence intervals. An exploratory multiple linear regression model with eGFR as the dependent variable and anti-HEV IgM status and time since transplantation as predictors was also fitted in the subset of patients with available data; the R² from this model should be interpreted as highly provisional given the small sample size. A pre-specified sensitivity analysis was conducted by excluding borderline serological results and repeating all the primary comparisons.

Because of the large number of statistical tests in a small cohort, no formal adjustment for multiple testing was applied; instead, p-values were reported as descriptive measures, and emphasis was placed on effect sizes and the consistency of patterns across related endpoints rather than on any single threshold for statistical significance. A two-sided p-value < 0.05 was considered statistically significant, and p values between 0.05 and 0.10 were interpreted as indicating a trend, but all inferential results should be regarded as hypothesis-generating rather than confirmatory. All analyses were performed using Python 3.12 (SciPy 1.12; scikit-learn 1.4).

## 3 Results

### 3.1 Patient characteristics and prevalence of HEV markers

A total of 25 kidney transplant recipients were included in this study. The mean age was 48.9 ± 14.4 years (range 24–75); 12 (48%) were female. The median time since transplantation was 13.0 years (mean 12.4 ± 7.0; range, 2–31 years). eGFR data were available in 15 of 25 patients (60%), with a median eGFR of 51.1 mL/min/1.73 m² (range 13.2–89.0); the remaining 10 patients did not have a measurement recorded during the study period, reflecting the retrospective design. Baseline liver enzyme values were available for 24 patients: median ALT, 19.0 U/L (range 9–193 U/L); median AST, 22.0 U/L (range 15–104 U/L); median GGT, 26.5 U/L (range 11–145 U/L); and median ALP, 95.0 U/L (range, 58–232 U/L). The UACR was available in 21 patients, with a median of 3.5 mg/g (range 0.3–236.9). Clinician-rated graft function was classified as worsening in 11 and better in 7 patients, while 7 (28%) were not formally classified and were excluded from categorical graft function analyses (**Table 1**).

Anti-HEV antibodies were detected in 19 of the 25 patients (76%). Among these, 4 patients (16%) had isolated anti-HEV IgM positivity (IgM positive with negative IgG), 6 patients (24%) had concurrent anti-HEV IgM and IgG positivity, and 9 patients (36%) had isolated anti-HEV IgG positivity without detectable IgM. The remaining 6 patients (24%) were negative for both anti-HEV IgM and IgG antibodies. None of the patients had isolated borderline results for either isotype. HEV RNA was detected in the serum of 2 patients (8%), with concurrent stool RNA positivity confirmed in both cases, yielding 2 RNA-positive patients in total (8%). One viremic patient had isolated HEV infection and fulfilled criteria for chronic HEV infection (detectable HEV RNA on two occasions over a six-month period); the other had HCV/HEV co-infection with active viremia for both pathogens documented over six months. The patient with isolated chronic HEV infection was treated with ribavirin (400 mg/day, dose-adjusted for renal function) and achieved sustained virological response, confirmed as undetectable HEV RNA in serum on two consecutive samples at least four weeks apart after completion of therapy, with concurrent normalisation of liver enzymes. The patient with HCV/HEV co-infection could not receive ribavirin; treatment with glecaprevir/pibrentasvir, initiated primarily for HCV, resulted in clearance of HEV RNA from serum, although HEV RNA remained detectable in stool at the last available follow-up. Both patients had HEV RNA confirmed on at least two occasions over a six-month period, fulfilling criteria for chronic HEV infection prior to treatment. HEV genotyping was not performed, as this assay was not available at our laboratory during the study period; based on the endemic profile of the region, HEV genotype 3 infection is the most probable, consistent with all published European transplant series.

Anti-HEV seropositivity (IgM and/or IgG reactivity) was observed in 19 of 25 patients (76%) and was distributed across various etiologies of end-stage kidney disease, including glomerulonephritis, diabetic nephropathy, IgA nephropathy, vesicoureteral reflux, systemic lupus erythematosus, and polycystic kidney disease, without a clear predominance of any single diagnostic category.

### 3.2 Sex, age, and HEV markers

There was no significant association between sex and HEV IgG (OR = 2.25; Fisher’s exact p = 0.43) or HEV IgM positivity (OR = 1.12; p = 1.00). Similarly, age did not differ significantly between HEV IgG-positive and -negative patients.

### 3.3 Three-group analysis by anti-HEV IgG category

When patients were stratified into three groups by anti-HEV IgG status (positive, borderline, negative), no statistically significant differences were observed in eGFR (median 54.7, 54.3 and 27.6 mL/min/1.73 m², respectively; Kruskal-Wallis p = 0.42), ALT (median 23.0, 18.5 and 19.0 U/L; p = 0.24), GGT (median 32.0, 26.5 and 20.5 U/L; p = 0.69), or ALP (median 89.0, 119.0 and 92.0 U/L; p = 0.27). A trend toward higher AST values was noted across IgG categories (median 25.0, 22.5 and 19.0 U/L, respectively; p = 0.07). Similarly, the UACR showed numerically higher values in IgG-negative patients (median 27.2 mg/g) than in the IgG-positive (2.9) and borderline (2.6) groups, but the difference was not statistically significant (p = 0.37).

### 3.4 HEV IgG serostatus and allograft parameters

In the primary binary analysis (IgG-positive vs. non-positive), there was no significant difference in eGFR (median 54.7 vs 48.4 mL/min/1.73 m²; p = 0.77). ALT values tended to be higher in IgG-positive patients (median 23.0 vs 19.0 U/L; p = 0.10; Cliff’s δ = 0.41, large effect), while AST was significantly higher in IgG-positive patients (median 25.0 vs 20.5 U/L; p = 0.03). No significant differences were observed in GGT (p = 0.48), ALP (p = 0.32), and UACR (median 2.9 vs 5.3 mg/g; p = 0.33). When graft function was analysed as a binary outcome (worsening vs. better, excluding patients without a formal rating), HEV IgG positivity was not significantly associated with worsening graft function (OR = 1.43; 95% CI 0.13–15.3; Fisher’s exact p = 1.00; **Table 2**).

**Table 2:** Clinical and biochemical parameters stratified by anti-HEV IgG status.

Parameter	IgG+ (n = 10)	IgG– (n = 15)	P value
eGFR, mL/min/1.73 m², median*	54.7 (n = 5)	48.4 (n = 10)	0.77
ALT, U/L, median	23.0	19.0	0.10
AST, U/L, median	25.0	20.5	0.03
GGT, U/L, median	32.0	25.0	0.48
ALP, U/L, median	89.0	103.0	0.32
UACR, mg/g, median†	2.9 (n = 8)	5.3 (n = 13)	0.33
Worsening graft function, n/N (%)‡	4/6 (67)	7/12 (58)	1.00 (OR 1.43; 95% CI 0.13–15.3)

*eGFR available in 15; †UACR in 21; ‡18 with rated graft fn.

### 3.5 HEV IgM serostatus and allograft function

Anti-HEV IgM positivity was associated with a significantly lower eGFR (median 35.5 vs 57.5 mL/min/1.73 m²; Mann–Whitney p = 0.018; Cliff’s δ = 0.74, large effect). This was the most consistent signal observed in the present exploratory analysis, though it should be interpreted with caution given the small sample size and the availability of eGFR data in only a subset of patients. IgM-positive patients also showed a trend toward higher ALT (median 23.5 vs 18.5 U/L; p = 0.07; Cliff’s δ = 0.47, large effect). No significant differences were observed in AST (p = 0.37), GGT (p = 0.26), ALP (p = 0.69), or UACR (p = 0.64).

When graft function was analysed as a binary outcome, the proportion of patients classified as having worsening graft function was markedly higher among IgM-positive patients (6/8; 75%) than among IgM-negative patients (5/10; 50%), although this difference did not reach statistical significance (Fisher’s exact p = 0.37).

HEV IgM positivity was the strongest individual predictor of worsening graft function (OR = 3.00; 95% CI 0.37–24.5), exceeding the effect of IgG positivity (OR = 1.43; 95% CI 0.13–15.3), age (OR = 1.01 per year), and time since transplantation (OR = 1.05 per year; **Table 3**). The wide confidence intervals reflect the limited number of outcome events and should be interpreted accordingly.

**Table 3:** Clinical and biochemical parameters stratified by anti-HEV IgM status.

Parameter	IgM+ (n = 8)	IgM– (n = 17)	P value
eGFR, mL/min/1.73 m², median*	35.5 (n = 6)	57.5 (n = 9)	0.018
ALT, U/L, median	23.5	18.5	0.07
AST, U/L, median	24.0	21.0	0.37 (OR 3.00; 95% CI 0.37–24.5)
GGT, U/L, median	30.0	25.0	0.26
ALP, U/L, median	102.0	94.0	0.69
UACR, mg/g, median†	3.9 (n = 6)	3.5 (n = 15)	0.64
Worsening graft function, n/N (%)‡	6/8 (75)	5/10 (50)	0.37

*eGFR available in 15; †UACR in 21; ‡18 with rated graft fn.

### 3.6 Time from transplantation and HEV serostatus

There was no significant correlation between the time since transplantation and HEV IgG serostatus (Spearman’s r = −0.09; p = 0.69). Conversely, anti-HEV IgM positivity was positively correlated with time since transplantation (Spearman r = 0.44; p = 0.035). No significant correlation was observed between the time since transplantation and IgG titres (r = −0.072; p = 0.79).

### 3.7 Exploratory regression analyses

In an exploratory multiple linear regression model with eGFR as the dependent variable and anti-HEV IgM status and time since transplantation as predictors (n = 15), anti-HEV IgM positivity was associated with a reduction of approximately 29.8 mL/min/1.73 m² in eGFR (coefficient −29.82), while time since transplantation showed a modest positive coefficient (1.15 mL/min/1.73 m² per year). The model explained approximately 50% of the variance in eGFR (R² = 0.50). Given the small sample (n = 15, 2 predictors), this model is strictly exploratory and hypothesis-generating; the R² and coefficient estimates should not be over-interpreted.

In univariate logistic regression for worsening graft function, anti-HEV IgM was the strongest predictor (OR = 3.00; 95% CI 0.37–24.5), followed by time since transplantation (OR = 1.05 per year), IgG positivity (OR = 1.43; 95% CI 0.13–15.3), and age (OR = 1.01 per year). However, owing to the limited number of outcome events, multivariable logistic regression was not performed.

### 3.8 Sensitivity analysis

When borderline IgG results were excluded and the analysis was restricted to IgG-positive (n = 10) versus IgG-negative (n = 6) patients only, AST remained significantly higher in IgG-positive patients (median 25.0 vs 19.0 U/L; p = 0.045), supporting the consistency of this finding across serological definitions. The differences in ALT (p = 0.28), eGFR (p = 0.29), GGT (p = 0.55), ALP (p = 0.96), and UACR (p = 0.20) remained non-significant, but showed similar directional patterns regardless of the definitions of HEV IgG seropositivity.

### 3.9 Composite HEV exposure and graft function

When a composite variable was defined as any positive HEV marker (IgG or IgM), 16 patients (64%) were classified as having evidence of HEV exposure. The odds of worsening graft function were higher in HEV-exposed patients, but this was not statistically significant (OR = 2.00; Fisher exact p = 0.63).

## 4 Discussion

In this single-centre cross-sectional study of 25 kidney transplant recipients, we observed a notably high prevalence of HEV markers, with 40% anti-HEV IgG and 32% anti-HEV IgM seropositivity. In 2 of the 25 patients (8%), active HEV replication was confirmed by detectable HEV RNA, documenting ongoing viral infection in this cohort. The anti-HEV IgG seroprevalence of 40% observed in our cohort was considerably higher than that reported in most kidney transplant populations in Western Europe and the United States (12). In a large US single-centre prospective study by Lim et al., anti-HEV IgG was detected in 18% of 244 kidney transplant candidates, increasing to 26% in the first-year post-transplant, although HEV RNA remained undetectable throughout follow-up (13). In a pre-transplant evaluation of 201 kidney candidates at the Mayo Clinic, Unzueta et al. reported an anti-HEV IgG seroprevalence of only 8.5%, with no evidence of active viremia (14). A French study of 700 kidney and liver transplant candidates reported a seroprevalence of 12.7% (15). In Serbia, a population-based study in blood donors reported that 15% tested positive for anti-HEV IgG (16).

An exploratory association was observed between anti-HEV IgM positivity and lower eGFR in kidney transplant recipients, accompanied by a large effect size in non-parametric analyses. This finding is hypothesis-generating and must be interpreted with considerable caution in the context of the small sample size, the availability of eGFR data in only 15 of 25 patients, only two cases of RNA-confirmed chronic HEV infection, and the cross-sectional design, which precludes causal inference. The wide confidence intervals around all reported effect estimates reflect substantial statistical uncertainty, and no causal relationship between HEV and allograft dysfunction can be inferred from these data. We cannot determine from the current data whether IgM positivity reflects a direct renal effect of HEV, immune-mediated graft injury, or confounding by unmeasured variables. This pattern was paralleled by a higher proportion of IgM-positive patients with clinician-rated worsening graft function and by exploratory regression models in which IgM positivity remained associated with lower eGFR, although estimates were imprecise and based on a small subset with complete data. While past HEV exposure captured by anti-HEV IgG seropositivity was not associated with current graft function, recent or ongoing infection reflected by anti-HEV IgM positivity was associated with a numerically lower eGFR, a higher proportion of patients with clinically worsening graft function, and a positive correlation with time since transplantation. These observations are compatible with the hypothesis that HEV may be a contributing factor in late graft dysfunction in some patients, but this hypothesis requires confirmation in larger prospective studies before any clinical conclusions can be drawn. Regarding the interpretation of anti-HEV IgM positivity in clinical practice: serology alone is insufficient to confirm active HEV infection in immunosuppressed patients. Isolated IgM positivity may reflect early acute infection, an atypical antibody response, or a false-positive result, and active infection requires HEV RNA confirmation. In the present cohort, serum RNA was negative in the majority of IgM-positive patients, so active systemic viremia was not confirmed in most. We therefore do not propose anti-HEV IgM as a diagnostic or management-changing marker. Rather, IgM positivity may serve as a signal warranting reflexive HEV RNA testing, particularly in patients with unexplained graft dysfunction or transaminase elevation. The current data support targeted HEV evaluation in such patients but do not establish that IgM positivity alone should change clinical management.

In our cohort, anti-HEV IgM positivity was positively correlated with time since transplantation (Spearman r = 0.44; p = 0.035), suggesting that late post-transplant HEV acquisition or reactivation may be favoured by cumulative immunosuppression, progressive immunosenescence, or both. This observation is consistent with the findings of Lim et al., who demonstrated that HEV seroconversion was associated with the degree of immunosuppression, as evidenced by a significant association between concomitant BK virus and cytomegalovirus viremia and HEV seroconversion, as well as diabetes, which were identified as independent risk factors (13). Similarly, Unzueta et al. found that older age (≥60 years) was the sole independent predictor of HEV IgG seropositivity in their pretransplant cohort, consistent with the hypothesis of cumulative lifetime HEV exposure (14). These data collectively suggest that HEV testing should not be limited to the early post-transplant period but should be considered at any point when unexplained graft dysfunction or mild transaminase elevations are observed, particularly in long-term recipients.

In a landmark study, Schmitz et al. at Hannover Medical School identified HEV RNA within tubular epithelial cells of renal allografts using advanced microscopy and molecular techniques in transplanted patients with contemporaneous HEV viremia (16). In our cohort, active HEV infection was documented in two patients by detectable HEV RNA, representing the only cases with confirmed ongoing viral replication. Although renal biopsy with HEV-specific tissue staining was not performed, it is conceivable that subclinical renal HEV tropism, as described in recent reports, may contribute to the lower eGFR observed in IgM-positive patients (17, 18).

The risk of chronicity in HEV-infected transplant recipients has been well established (19). Matias et al. recently described two kidney transplant recipients with chronic HEV genotype 3 infection (subtypes 3c and 3ra), both identified during routine follow-up because of asymptomatic but persistent elevations in liver enzymes (20). In both cases, reduction of immunosuppression alone failed to clear the virus. The patient with isolated chronic HEV achieved sustained virological response after a course of ribavirin. The patient with HCV/HEV co-infection, who could not receive ribavirin, was treated with glecaprevir/pibrentasvir and achieved HEV RNA clearance from serum, though stool shedding persisted at last follow-up. These cases illustrate the clinical heterogeneity of HEV management in kidney transplant recipients and the potential utility of direct-acting antivirals in patients with contraindications to ribavirin. They also underscore the importance of early detection to allow timely intervention before the development of progressive fibrosis and highlight that stool RNA testing may remain positive after serum clearance.

While anti-HEV IgG positivity (remote exposure) was not associated with eGFR differences in our study, IgG-positive patients had significantly higher AST levels (p = 0.03) and showed a trend toward higher ALT levels (p = 0.10), suggesting subclinical hepatocellular involvement even in patients without active viremia. The significant correlation between IgG titre and AST (r = 0.595; p = 0.012) further supports this observation and raises the possibility that quantitative serological assessment may provide clinical information beyond binary serostatus. These findings are consistent with the known pattern of mild, often asymptomatic transaminase elevations in HEV-infected transplant recipients, where ALT elevations are typically modest (100–300 IU/L) and can be easily overlooked in the post-transplant setting where hepatotoxic drug effects are common (7).

Our findings suggest that post-transplant screening may have an even greater yield, particularly in populations with higher baseline HEV seroprevalence. Several groups have proposed screening strategies for HEV in solid organ transplant recipients, focusing on targeted testing in patients with unexplained liver enzyme elevation or graft dysfunction (21, 22). Such screening could be targeted to patients with unexplained graft dysfunction, mild transaminase elevations, or both, particularly in endemic regions.

The potential link between HEV infection and glomerulonephritis in kidney transplant recipients deserves particular attention. Cases have been reported where HEV infection may serve as a trigger for immune-complex-mediated glomerular disease, including relapses of IgA nephropathy in HEV-infected patients (23). Cryoglobulinemia and glomerulonephritis have been linked to HEV infection in several studies (24).

In summary, this small single-centre pilot study of kidney transplant recipients in a HEV-endemic region identified a high burden of HEV serological markers and an exploratory signal linking anti-HEV IgM positivity with lower eGFR and clinician-rated worsening graft function. These findings add to the growing body of evidence that HEV infection may be associated with clinically relevant renal and extrahepatic manifestations in immunosuppressed hosts, highlighting the need to maintain a high index of suspicion of HEV in transplant recipients with subtle liver enzyme abnormalities or unexplained late allograft dysfunction. The question of donor-derived HEV transmission is directly relevant to the transplant setting. At the time of this study, routine HEV screening of deceased organ donors was not standard practice in Serbia, and donor HEV serology and RNA data were not available for the present cohort; donor-derived infection therefore cannot be assessed retrospectively. HEV donor screening policies vary considerably across Europe, and the recently published study by Slepčanová et al., reporting an anti-HEV IgG seroprevalence of 49.3% among deceased organ donors in the Czech Republic, underscores the potential scale of undetected donor HEV exposure in the Central and Eastern European region (25). These data, together with our findings, support the need to evaluate systematic HEV screening in the transplant donor–recipient dyad in endemic regions of South-Eastern Europe.

## 5 Limitations

This study has several important limitations. It was conducted in a single centre with a small sample size (n = 25), and eGFR data, as well as clinician-rated allograft status, were not available for all participants, which limits statistical power and may have introduced selection bias. The cross-sectional design precludes causal inference and does not allow us to distinguish transient from persistent HEV infection. We did not perform HEV genotyping or protocol biopsies to directly link viral status with histological lesions. HEV genotyping was not available at our laboratory during the study period; based on the regional epidemiological profile, HEV genotype 3 is the most probable. Bilirubin and synthetic liver function parameters (albumin, INR) were not systematically available. One patient had HCV/HEV co-infection, which may have independently contributed to liver enzyme elevations and graft dysfunction in that individual; systematic exclusion of HAV and HDV co-infection was not performed for the full cohort. CMV and EBV reactivation, which are common in transplant recipients and may influence both liver biochemistry and graft function, were not systematically assessed in this retrospective cohort and represent important unmeasured confounders. Additional parameters relevant to allograft phenotyping—including cystatin C, urinary sediment, donor-specific antibodies, rejection history, and renal biopsy findings—were not available. The clinician-rated categorisation of graft function was not based on pre-specified quantitative criteria and carries a risk of ascertainment bias, as clinicians were not blinded to HEV serological status. HEV testing was not part of a universal screening protocol during the study period, and potential enrichment of the sample for symptomatic or biochemically affected patients cannot be fully excluded. Donor HEV screening data were not available, so donor-derived transmission cannot be assessed. Confirmatory follow-up serology was not obtained for all IgM-positive patients. Extrahepatic manifestations of HEV, including neurological complications, were not prospectively assessed. Nevertheless, the convergence of several quantitative signals—high seroprevalence, a consistent IgM-associated decrement in eGFR with a large effect size, and the correlation between IgG titres and AST—provides a rationale for further investigation, while causality remains unestablished and confirmation in larger prospective studies is required before any conclusions about the clinical impact of HEV on kidney allograft function can be drawn.

## 6 Conclusion

In conclusion, kidney transplant recipients in our South-Eastern European cohort exhibited a high burden of HEV exposure and infection. In this exploratory pilot analysis, anti-HEV IgM positivity was associated with lower eGFR and a higher proportion of clinician-rated worsening graft function, while IgG seropositivity was associated with subclinical hepatic enzyme changes. These hypothesis-generating findings, derived from a small cohort with incomplete eGFR data and wide confidence intervals, do not permit causal inference but support targeted HEV evaluation, including HEV RNA testing, in kidney transplant recipients with unexplained graft dysfunction or mild transaminase elevation, particularly long-term recipients in endemic regions. Anti-HEV IgM should not be used as a standalone diagnostic or management-changing marker; RNA testing remains necessary to confirm active infection. Larger prospective, longitudinal studies with kidney biopsies are needed to define the renal impact of HEV, clarify causal pathways, and determine whether HEV testing should be integrated into routine transplant surveillance.

## Data Availability

The raw data supporting the conclusions of this article will be made available by the authors, without undue reservation.
